# Development of the Kunonga framework for operationalising approaches to health inequality and/or inequity evidence syntheses

**DOI:** 10.1186/s12889-025-25688-4

**Published:** 2025-11-22

**Authors:** Tafadzwa Patience Kunonga, Eugenie Evelynne Johnson, Pauline Addis, Elizabeth Westhead, Peter Bower, Barbara Hanratty, Dawn Craig

**Affiliations:** 1 Population Health Sciences Institute, Newcastle University, National Institute for Health and Care Research (NIHR) Policy Research Unit in Older People and Frailty/Healthy Ageing, Newcastle-Upon-Tyne, NE4 5PL UK; 2 Population Health Sciences Institute, Newcastle University, National Institute for Health and Care Research (NIHR) Innovation Observatory, Population Health Sciences Institute, Newcastle University, Newcastle Upon Tyne, NE4 5TG UK; 3 Population Health Sciences Institute, Biomedical Research Building, Newcastle University, Newcastle Upon Tyne, NE4 5PL UK; 4 School of Health Sciences, Faculty of Biology, Medicine and Health, The University of Manchester, National Institute for Health and Care Research (NIHR), The University of Mancheste, Manchester, M13 9PL UK

**Keywords:** Health inequality, Health inequity, Intersectionality, Evidence synthesis, Life-course perspective

## Abstract

**Background:**

Health inequalities and inequities are shaped by intersecting social determinants and cumulative life-course experiences. However, conventional evidence synthesis methods often lack the conceptual and analytical tools to capture this complexity. This limits their ability to inform inequality and/or inequity-sensitive policy and practice. In response, we developed a methodological framework to support the systematic integration of intersectionality and life-course perspectives into evidence synthesis.

**Methods:**

Framework development followed a three-phase process. First, a systematic review identified conceptual and operational limitations in existing synthesis methods that seek to address health inequality and/or inequity. Second, semi-structured discussions were conducted with eight experts in health inequalities and evidence synthesis to elicit practice-relevant insights aimed at addressing these gaps. Third, findings from both phases were synthesised using a modified framework analysis to construct a structured thematic model aligned with key stages of evidence syntheses (protocol development, data extraction, analysis and synthesis), informing the design of the methodological framework.

**Results:**

The Kunonga Framework offers practical tools and methodological guidance for integrating inequality and/or inequity considerations across protocol development, data extraction, analysis, and synthesis stages. It is underpinned by three core principles: (1) distinguishing between health inequality and health inequity to improve conceptual clarity; (2) applying intersectionality to examine how overlapping social disadvantages shape health outcomes; and (3) adopting a life-course perspective to assess how inequalities and inequities emerge, accumulate and evolve over time. The framework provides practical tools, including logic models, intersectionality-informed extraction template and life-stage mapping, to support implementation. A case study on ethnic inequalities in palliative care prescribing illustrated the framework’s feasibility and highlighted its potential to enhance analytical depth and policy relevance.

**Conclusions:**

The Kunonga Framework advances review methodology by translating intersectionality and life-course theory into practical guidance across protocol development, data extraction, analysis, and synthesis. It helps reviewers consider social complexity and change over time to understand for whom, how, and in what contexts interventions work. Although developed for inequality-focused reviews, future work should test the framework across different review types and methods and extend its principles of intersectionality and life-course to earlier stages, including searching and screening.

**Supplementary Information:**

The online version contains supplementary material available at 10.1186/s12889-025-25688-4.

## Background

Evidence synthesis involves systematically gathering, evaluating, and integrating existing research findings to provide a comprehensive and robust summary of available evidence on a particular topic [1]. It encompasses a range of methodologies including systematic reviews, meta-analyses and qualitative syntheses, depending on the nature of the research question [1]. Evidence synthesis plays a crucial role in driving evidence-based decision-making processes, particularly as policymakers increasingly direct their attention towards addressing issues of inequality and inequity [2].

The terms health inequality and health inequity are often used interchangeably, but their meanings vary across countries and disciplines [3]. In the United Kingdom, the public health community has long used health inequalities to describe systematic, avoidable, and unfair differences in health, essentially with the same meaning as health inequities [4]. In Canada and several other settings, inequities is the preferred term when referring to these normative, unjust differences [5]. Across many European countries, health inequities (or their direct equivalents) are more commonly used, and in some languages the two terms are indistinguishable because only a single word exists for both [5]. At the same time, health economists in the UK and across Europe often use health inequalities in a purely descriptive sense to denote measurable differences in health without implying unfairness. In the United States, the term health disparities is more widely used than either inequalities or inequities and tends to be less explicitly linked to moral or justice-based concepts of fairness [4]. For the purposes of this article, health inequality refers to differences in health status or determinants among individuals or populations, while health inequity specifically denotes unnecessary, avoidable and unjust differences in health [6]. Health inequalities remain a pressing issue globally, with outcome differences persisting across various demographic groups [7].

While evidence synthesis methodologies play a crucial role in informing healthcare policies and interventions, there are significant gaps in how they address the impacts of health inequalities and inequities [8]. As identified by a recently published systematic review, a range of frameworks have been developed to address aspects of equality and/or equity across health and care research [9]. These include, but are not limited to: the World Health Organization (WHO) conceptual framework on the Social Determinants of Health (SDOH), which distinguishes between structural and intermediary determinants [10]; the Health Equity Impact Assessment (HEIA), designed to assess potential differential impacts of policies or interventions [11]; the PROGRESS-Plus framework,^1^ which identifies key social factors contributing to health inequalities and/or inequities [12, 13]; and, more recently, the EQUALSS Guide Multiple framework,^2^ which supports equity priority-setting across domains [14]. However, while these frameworks are useful for identifying relevant socio-demographic factors, they were not originally developed to guide how inequality or inequity should be understood, interpreted or systematically analysed within evidence synthesis processes. This methodological gap was identified in our earlier systematic review [9], which highlighted the absence of integrated, operational guidance to consistently embed theoretically grounded equality and/or equity principles, such as intersectionality and life-course approaches, across all stages of the evidence synthesis process [9].

Specifically, the review found that factors contributing to inequality and/or inequity were often considered in isolation, with limited attention to how multiple factors intersect to shape health outcomes in complex, compounding ways [9]. Similarly, life-course approaches, which recognise how cumulative exposures and structural disadvantage across an individual’s lifespan influence health trajectories [15], remain underutilised in evidence synthesis. Yet, applying concepts such as intersectionality and life-course perspectives is essential to understanding how inequalities and/or inequities emerge and persist, a challenge often overlooked in current evidence syntheses. Their limited and fragmented application within evidence synthesis constrains the field’s capacity to support inequality and/or inequity-sensitive decision-making. There remains a need for a methodological framework that translates established theories of inequality and inequity into practical tools for conceptually coherent, equity-sensitive evidence synthesis.

### Research aim

To develop and present a structured approach for embedding theoretically grounded perspectives on health inequality and/or inequity within evidence syntheses that focus on, or include, inequality or inequity related objectives.

### Objectives

1. To synthesise expert insights into the conceptual, analytical, and practical challenges of addressing health inequality and/or inequity within evidence synthesis methodologies.
2. To construct the Kunonga Framework, an operational guide for integrating intersectionality and life-course perspectives throughout key stages of evidence synthesis.
3. To demonstrate the framework’s practical application and value through a case study on ethnic inequalities in palliative care prescribing.

## Methods

The development of the proposed framework followed a modified three-stage approach, drawing on recommended phases for methodological framework development [16].

### Phase 1: evidence mapping and problem framing

The first phase, as outlined in the introduction, involved conducting a comprehensive systematic review to assess existing approaches in inequalities/inequities-focused evidence synthesis. The methods and findings of this review have been reported previously [9], establishing a foundation for developing a more inclusive framework tailored to the complexities of health inequalities/inequities within evidence synthesis. Therefore, this paper will not elaborate further on these review findings but instead concentrates on the subsequent phases that build upon these insights to develop the proposed framework.

### Phase 2: expert knowledge elicitation

Phase 2 involved small-group key informant discussions with experts in health inequalities, inequities, and/or evidence synthesis to generate insights for methods guidance, addressing the gaps identified in Phase 1. While key informant approaches are typically applied in one-to-one formats [17, 18], we adapted the method to small-group settings to encourage interactive dialogue and peer-level reflection. This approach prioritised conceptual depth and targeted the insights of information-rich participants to examine methodological limitations and identify feasible strategies for integrating inequality and/or inequity considerations into evidence synthesis. As this phase involved expert consultation rather than primary research with patients or the public, it was approved by the Newcastle University Ethics Committee without detailed review (Ref: 61,394/2023). This phase was reported in line with the 32-item Consolidated Criteria for Reporting Qualitative Research (COREQ) checklist (Supplementary File 1) [19].

### Selection and recruitment of experts

Experts were selected based on their demonstrated contributions to their respective fields, evidenced by substantial peer-reviewed publications and other scholarly outputs. This criterion ensured that participants brought a depth of expertise and a history of impactful research, thereby enhancing the rigour and credibility of insights gathered for this study [17, 20]. The recruitment process aimed to ensure diversity of perspectives, including individuals from different sectors and countries. Thirteen experts were contacted via email, introducing the project, and inviting them to participate in the workshop. The email outlined the objectives, expected time commitment and the potential benefits of involvement. Interested experts were asked to confirm their participation, and a suitable date for the discussions was selected based on their availability.

### Pilot workshops

Two virtual pilot sessions were held with members of the Evidence Synthesis Group at Newcastle University (UK) to gather preliminary feedback and advice for the upcoming key informant discussions. These pilot sessions tested the interview content, activities, logistics and overall participant experience to ensure the effectiveness of the planned approach. Feedback from these sessions directly informed the development of the discussion questions used in the key informant discussions:

- What are the challenges or limitations faced in defining and consistently reporting health inequality and/or inequity within current evidence synthesis methodologies?
- What alternative methodologies or approaches could provide a more multidimensional synthesis of evidence on health inequalities/inequity, capturing a broader range of influencing factors beyond single variables like socioeconomic status?

### Key informant discussions

Three structured small-group discussions were conducted between March and July 2023. The discussions were led by TPK, an experienced evidence synthesis researcher and facilitated by another researcher (PA). Each interview was held online for approximately 90 min and involved at least two experts, allowing for dynamic discussions. The sessions began with a five-minute introduction, followed by a 10-min presentation of key findings from a case study to establish real-world context [9]. Participants then engaged in a 30-min brainstorming exercise. Another 10-min presentation of a second case study, followed by another 30-min brainstorming session [21]. The sessions closed with a summary and conclusion. An outline of the session agenda is presented in Supplementary File 2.

### Data collection

Sessions were conducted via Zoom and audio-recorded to capture the conversations and discussions. Recordings were accessible only to the two researchers who facilitated the sessions (TPK and PA), who transcribed and anonymised the data for analysis. For confidentiality, each participant received a unique identifier to attribute their quotations, ensuring that personal data protection protocols were maintained [22].

### Phase 3: synthesis and framework development

Qualitative data from the key informant discussions were analysed using approach adapted from the principles of framework analysis [23]. Drawing on methodological gaps identified in the review conducted in Phase1 [9], an initial coding framework was developed and applied deductively across transcripts. Two researchers (TPK and EW) independently read the transcripts, coded the data manually and organised them thematically to support comparison across topics. The analysis focused on extracting practice-relevant insights to inform the structure and content of the proposed framework. The final coding structure is presented in Supplementary File 3. The final themes were aligned with the stages of the evidence synthesis process: protocol development, data extraction and analysis, by researchers and decision-makers.

## Results of the key informant discussions

### Characteristics of key informants

Of the 13 originally contacted, eight experts participated in the key informant discussions. The final sample included six experts based in the UK and two based in Africa. Participants brought expertise from a range of domains, including public health, health economics, global health, and evidence methodology. Their roles spanned academia, policy advisory and applied research. Table 1 provides an overview of the participants’ areas of expertise and geographic affiliations.

**Table 1:** Characteristics of experts

Expert	Area of Expertise	Current Title	Country/Region	Disciplinary Background
1	Evidence synthesis	Professor of Health & Wellbeing Evidence	UK	Public Health/Evidence Synthesis
2	Evidence synthesis	Professor in Evidence Synthesis	UK	Information Science/Systematic Reviews
3	Health inequality	Professor of Health Inequalities	UK	Health Economics/Data Science
4	Evidence synthesis	Senior Lecturer in Evidence Synthesis	UK	Public Health/Evidence-Based Practice
5	Health inequality	Public Health Expert	Africa	Health Promotion/Social Determinants of Health
6	Health inequality	Health Economist	UK	Health Economics
7	Health equity	Statistical Advisor	UK	Epidemiology/Public Health
8	Health inequality and synthesis	Emeritus Professor	Africa	Evidence Synthesis

### Key methodological challenges identified by experts

The discussions generated insights into the practical and conceptual challenges of integrating inequality and/or inequity considerations into evidence synthesis. Experts reflected on the complexities of applying key principles in diverse contexts and offered methodological strategies to address these challenges. Most examples discussed by experts related to quantitative and mixed-method reviews, reflecting their experience and the focus of current methodological literature.

### Conceptual and terminological ambiguity

A persistent challenge identified by experts was the lack of conceptual clarity and terminological consistency surrounding the terms health inequality and health inequity. While these distinctions are theoretically established, experts noted that in practice, particularly in secondary data analysis, these terms are often conflated or applied inconsistently, with implications for study inclusion, framing, and interpretation. One expert noted the challenge of distinguishing these concepts when working with pre-existing data.

“*When doing a study of real data, the theoretical difference is not always apparent when using secondary data as to whether it relates to inequality or inequity… but generally one should try to use them correctly*.” [Expert 7]

In addition, the meanings of inequality and inequity may vary across languages and cultures, leading to different interpretations and understandings. Translating these terms accurately while maintaining their subtle distinctions can be challenging, potentially resulting in miscommunication and ambiguity.

“…*especially when English is not their first language, the translation of inequality and inequity isn’t as clear as it is in English. Internationally, there is a tendency to use inequity, when they mean inequality*.” [Expert 4]

*"In French, they are pretty much interchangeable terms… in Finland, they just use equity."* [Expert 3]

Even within the same language, different researchers may use the terms interchangeably or use other terms to define similar concepts.

“…*there is another term that is used quite a lot, particularly in North America, and that is disparity*.” [Expert 8]

The implication is that lack of definitional clarity not only undermines analytic precision but risks misclassification of studies, inconsistent application of inclusion criteria and miscommunication within international collaborations. Experts advocated for explicit articulation of these terms at the protocol stage, alongside guidance on how to operationalise them within synthesis.

### Operationalising intersectionality in evidence synthesis

Experts expressed widespread support for intersectionality as a necessary theoretical lens to understand how structural disadvantage accumulates across multiple dimensions. However, they also emphasised the disconnect between this conceptual ambition and the methodological realities of synthesis. Key barriers included the absence of disaggregated primary data on key socio-demographic factors (e.g. ethnicity, gender identity and socioeconomic status), lack of transparency in subgroup reporting, and limited statistical power for intersectional subgroup analysis.

*“...and if it’s not being collected, it’s not being reported, and you can’t really consider it as a reviewer.*" [Expert 2]

Importantly, experts linked these limitations to upstream challenges in primary research design and data governance. For example, one expert highlighted how data protection concerns may preclude collection of variables critical to intersectional analysis.

“*Basically, I don't think you could gather data from a study what I know. You can't gather data in the hope that future studies might be able to access that data*” [Expert 6]

Another identified tension was the lack of shared understanding about whether intersectionality is a statistical approach (e.g. interaction testing) or a critical interpretive lens. While some experts equated it with methodological tools (e.g. multivariable regression), others emphasised its epistemological role in reshaping what questions are asked, which populations are visible, and how absence of evidence is interpreted.

“*You know, it's not just one factor which is causing that particular outcome that there are other factors that may need to play, interact and so having that lens in itself is a useful thing”* [Expert 1]

One expert noted that qualitative research has an important role to play in addressing these challenges, particularly where quantitative data is insufficient. Qualitative evidence can surface the lived experiences of intersecting disadvantage, give voice to underrepresented groups, and offer unique insights into mechanisms of inequality and/or inequity that are not easily captured through standardised data.

Experts agreed that intersectionality is essential for understanding compounded disadvantage but difficult to apply in evidence synthesis. They called for better disaggregated data, clearer guidance on its analytical and interpretive use, and greater inclusion of qualitative evidence.

### Limited integration of life-course perspectives

The third challenge related to the limited integration of a life-course perspective in most evidence synthesis work. Experts observed that many reviews are shaped by the availability of cross-sectional data, which tends to focus on immediate factors affecting health. As a result, it can be difficult to capture how disadvantage builds up over time. This limitation was seen as restricting the ability of syntheses to explore the deeper, long-term pathways that contribute to health inequalities and/or inequities:

"...*because if you're showing that the resources, for example available to black children in the USA is on average far less...then you see that no, it's not, it's not genetic, it's a lack of resources at the in the very earliest days of life. You look at maternal or child mortality and so on. You can go right back to the start and then follow it through to adult life...*” [Expert 8]

The limited use of a life-course perspective was seen as a missed opportunity to understand how health outcomes are shaped by experiences across a person’s life. A more holistic view, tracing key transitions from childhood to adulthood, was seen as essential.

*"... what you need to do is take a holistic approach to social determinants... try to identify factors across the life-course.”* [Expert 5]

Experts also linked the life-course perspective to proportionate universalism, suggesting that individuals who have experienced greater disadvantage over time may require tailored or intensified interventions. However, few reviews were seen to incorporate this principle systematically.

"... *and that, I think, brings me to a term...which is I think even more controversial than the term inequity, which is proportionate, universalism which means if someone has had a lifetime of deprivation... you need greater effort to have an effect on their health outcomes.*" [Expert 6]

Experts noted that limited use of life-course perspectives hinders understanding of how disadvantage accumulates over time. They recommended integrating life-course thinking more systematically to reveal long-term pathways and support proportionate universalism.

### The Kunonga Framework

The Kunonga Framework was developed in response to methodological gaps and challenges identified through a prior systematic review [9], and expert consultation. Designed for evidence syntheses that focus on, or include, health inequality and/or inequity objectives, it targets the protocol development, data extraction, analysis, and synthesis stages, identified through expert consultation as areas where such considerations are often underdeveloped. Table 2 outlines the core components of the framework, providing practical guidance to improve conceptual clarity and to apply intersectionality and life-course perspectives across these stages.

**Table 2:** The Kunonga Framework: operationalising inequality and/or inequity-focused approaches in evidence synthesis

Stage	Component	Practical Guidance
Protocol Stage	Define Key Terms and Scope	1. Explicitly state whether the review focuses on inequality, inequity, or both 2. Provide clear definitions and examples to support inclusion/exclusion criteria 3. Clarify the conceptual focus to guide data extraction and interpretation 4. Involve interest-holders and public contributors early to reflect lived experience [24, 25]
	Frameworks and Theoretical Approach Selection	1. Identify and justify the relevant framework that will guide the review (e.g. PROGRESS-Plus, SDOH, HEIA, EQUALSS GUIDE Multiple) [10–12, 14] 2. Ensure alignment between framework, definitions, and review objectives 3. Justify use of intersectionality and/or life-course approaches depending on the review scope
	Developing a Logic Model	1. Create a visual pathway of how intersecting social determinants and temporal factors influence health outcomes [27] 2. Identify moderators (e.g. gender, policy context) and mediators (e.g. health literacy, access to care) [28] 3. Use to guide extraction strategy, subgroup selection, and theory-driven synthesis
Data Extraction Stage	Mapping Data	1. Capture disaggregated data on social stratifiers (e.g. ethnicity, gender, or disability) and contextual variables 2. Use an intersectionality informed extraction table to record how characteristics are combined or analysed together 3. Extract presence/absence of subgroup reporting and interpretation (e.g. structural vs individual explanations) 4. Note whether primary studies explicitly mention inequality/inequity and whether they include equity-related recommendations or policy implications 5. Map life-course variables: age at exposure, timing of interventions, key transitions (e.g. retirement, caregiving) [33] 6. Record missing data or absence of stratifiers as analytically meaningful
Analysis Stage	Intersectionality	1. Examine overlapping identities and power structures (not just additive factors) 2. Where possible, apply subgroup meta-analysis or regression models with interaction terms 3. Use MAIHDA or IPD where data allow for advanced intersectional analysis [34, 35] 4. When appraising included studies, assess whether analyses used theory-driven, minimal adjustment sets and avoided both over-adjustment (controlling for too many variables) and under-adjustment (omitting key factors) [36, 37] 5. For health-service use outcomes, consider whether analyses accounted for differences between socio-economic groups in clinical need to distinguish unfair inequalities or inequities from legitimate variation [38] 6. In qualitative or mixed-methods reviews, synthesise narrative findings to examine intersecting disadvantage 7. Note the depth of intersectionality in included studies (descriptive vs analytical use)
	Life-course Analysis	1. Code life stages (e.g. early childhood, midlife, old age) to explore cumulative and transitional effects [33] 2. Apply life-course models [33]: a) Critical period model (early life impact) b) Accumulation model (sustained disadvantage) 3. Assess timing of exposure and outcome measurement 4. Reflect on evidence gaps in temporal coverage (e.g. midlife underrepresented) 5. Use qualitative insights (e.g. life narratives, contextual framing, timing of exposures) can be used to supplement missing quantitative data
Synthesis Stage	Integrating and interpreting findings through an inequality/inequity lens	1. Integrate quantitative and qualitative evidence to explain mechanisms and context underlying observed differences [40, 41] 2. Examine consistency of patterns across intersections and settings; distinguish structural drivers from methodological artefacts [42] 3. Highlight data gaps (e.g., lack of disaggregation, small samples) and state how they constrain synthesis [41] 4. Assess confidence transparently (e.g., GRADE-CERQual; GRADE with equity considerations) and report implications for disadvantaged groups [43, 44] 5. Formulate inequality/inequity-relevant conclusions/recommendations for policy and practice, linked to the strength and transferability of the evidence

Key: PROGRESS Plus: Place of residence, Race/ethnicity, Occupation, Gender, Religion, Education, Socioeconomic status, Social capital, plus additional factors such as personal characteristics, features of relationships, and time-dependent relationships

*WHO SDOH* World Health Organization conceptual framework on the Social Determinants of Health, *HEIA* Health Equity Impact Assessment, *EQUALSS GUIDE *Multiple framework: Ethnicity and race, Qualifications and education, Underserved area, Age, Language and religion, Sex, Sexual orientation, Gender identification, Underrepresented groups (inclusion groups), Income and wealth, Disability (physical, mental and learning), Employment and occupation, and Multiple disadvantages, *PRISMA-S* Preferred Reporting Items for Systematic reviews and Meta-Analyses literature search extension, *MAIHDA* Multilevel Analysis of Individual Heterogeneity and Discriminatory Accuracy (MAIHDA), *IPD* Individual Participant Data, *GRADE* Grading of Recommendations Assessment, Development and Evaluation, *GRADE-CERQual* Grading of Recommendations Assessment, Development and Evaluation—Confidence in the Evidence from Reviews of Qualitative Research

### Protocol stage

#### Define key terms and scope

In addition to standard scope-setting practices, this stage requires that reviewers clarify whether the primary focus is on health inequality and/or health inequity, establishing explicit definitions and examples to guide the eligibility criteria. This approach complements existing frameworks by adding specificity, thereby enhancing alignment with the review’s objectives and clarifying priorities for analysis [13]. This stage should include structured involvement of interest-holders, defined as people and groups with legitimate interests in the review, such as those affected by or responsible for decisions informed by research, including clinical experts, public contributors, policymakers, researchers, and community representatives particularly those from structurally disadvantaged or underrepresented groups [24, 25]. Early engagement helps ensure the review reflects diverse lived experiences and social contexts [25]. It supports the identification of meaningful inequalities and/or inequities, relevant subgroups, culturally sensitive language, and an ethically grounded analytical lens. This aligns with inclusive research guidance such as the National Institute for Health and Care Research—Innovations in Clinical Trial Design and Delivery for the Under-served (NIHR-INCLUDE) [26].

#### Frameworks and theoretical approach selection

At this stage, reviewers should identify and justify the inequality and/or inequity framework or theoretical model that will guide their review. Depending on the review’s scope, focus and population, this may include established tools such as the SDOH, PROGRESS-Plus, HEIA, or the EQUALSS GUIDE Multiple framework for setting equality and/or equity priorities [10–12, 14]. The chosen framework should align with whether the review addresses health inequality, health inequity, or both, and inform eligibility criteria, data extraction and analysis plans.

#### Developing a logic model

Developing a logic model at the protocol stage offers a structured and transparent way to integrate inequality and/or inequity considerations throughout the synthesis process [27]. Rather than functioning as a static illustration, the logic model should be treated as a dynamic analytical tool that links theory to empirical strategy. It maps the hypothesised pathways through which social identities (e.g. gender, or ethnicity), structural determinants (e.g. housing insecurity, policy environment), and temporal exposures across the life-course (e.g. early-life adversity, transition into retirement) shape differential health outcomes and responses to interventions [27]. These factors may play different analytical roles within the model. Specifically, some may act as moderators, influencing the strength or direction of the relationship between an intervention and an outcome (e.g. socioeconomic status altering the effectiveness of a prevention programme). Others may function as mediators, explaining the mechanisms through which an intervention leads to an outcome (e.g. trust or health literacy mediating the link between provider communication and adherence) [28]. The same factor may play different roles depending on the review context. Articulating these hypothesised relationships early, the logic model may help reviewers make informed decisions about inclusion criteria, subgroup analyses, and what data should be extracted.

### Data extraction stage

The data extraction process must move beyond descriptive cataloguing of socio-demographic characteristics and towards actively capturing how these characteristics intersect, how mechanisms of inequality and/or inequity operate, and how broader contextual factors influence observed outcomes. We recommend that reviewers develop an intersectionality-informed extraction table, in addition to standard data extraction procedures, to systematically capture factors related to inequality and/or inequity. This approach is grounded in the foundational work on intersectionality and its application in health equity research [29, 30]. The table could include a range of core domains. First, reviewers should extract whether social stratifiers (e.g. from SDOH, PROGRESS-Plus, HEIA, or the EQUALSS GUIDE Multiple) are reported in primary studies and, crucially, whether these are analysed in isolation or in combination (e.g. low-income older migrants) [10–12, 14]. Reviewers should also note whether primary studies explicitly refer to inequality and/or inequity in their objectives, analyses, or conclusions, and whether they include equity-related recommendations or policy implications. Second, reviewers should extract whether outcomes are reported in a disaggregated manner for relevant subgroups or intersectional combinations and whether statistical interaction terms are used to explore compounded or synergistic effects. The interpretive treatment of observed differences could also be recorded, that is, whether the study authors contextualise differences in terms of systemic or structural inequities (e.g. access, discrimination, policy context), or attribute them to individual behaviours or cultural norms [31]. Where logic models have been developed at the protocol stage, they should inform the extraction of variables hypothesised to function as moderators or mediators [28]. Contextual information, including the type of health system, geographic region, and socio-political environment, should also be extracted, as these features are likely to shape both intervention effects and the experience of inequality [32]. A life-course perspective could also be integrated at this stage, through the extraction of timing variables (e.g. age at exposure, intervention duration) and transitional life events (e.g. retirement, caregiving responsibilities) [33]. Finally, reviewers should record patterns of underreporting or omission (e.g. absence of religion), as these gaps are analytically important and should inform later stages of synthesis. Where disaggregated data or statistical estimates are unavailable, reviewers should extract narrative descriptions or author observations to ensure such studies can still contribute to the synthesis. Using interpretation and context-based reasoning in these cases can still offer valuable insights into how and why outcomes vary, instead of leaving those studies out [32]. This approach aligns with calls to systematically extract contextual and mechanistic detail, enabling reviewers to ask not just what works, but for whom, in what context, and why [32]. A template of this table is provided in Supplementary File 4. Reviewers are encouraged to adapt the template to fit the nature of their review. Used effectively, this approach should allow reviewers to synthesise findings not only across different identities, but also across the structural conditions that produce and sustain inequity.

### Analysis stage

#### Intersectionality

To support intersectionality-informed analysis, reviewers should avoid examining social factors in isolation or treating them as simply additive. Instead, they should explore how these factors interact and overlap to shape health outcomes in more complex and meaningful ways [29]. Where quantitative data allow, reviewers may conduct subgroup meta-analyses comparing outcomes across different intersections or apply meta-regression to examine whether study-level characteristics such as participant demographics moderate intervention effects. More advanced methods such as Multilevel Analysis of Individual Heterogeneity and Discriminatory Accuracy (MAIHDA) offer a means of modelling between and within group variation across intersecting identities while maintaining statistical robustness [34]. This is particularly valuable because it preserves within-group variability and avoids the pitfalls of dichotomous subgrouping, allowing more accurate estimation of compounded disadvantage [34]. Where individual participant data (IPD) are available, reviewers can model interaction terms directly to test whether outcomes vary significantly across intersectional groupings (e.g. comparing low-income ethnic minority women with high-income white men) [35]. These methods can help reveal whether the combined effect of multiple social factors differs from their individual effects [29]. These methods reduce some limitations of traditional subgroup analysis but do not automatically prevent over-adjustment, for example, when studies control for factors that are part of the causal pathway, which can control away real effects [36, 37]. When appraising included studies, reviewers should assess whether analyses were guided by clear causal reasoning and whether they used appropriate, minimal adjustment sets. They should be alert to both over-adjustment (controlling for too many variables) and under-adjustment (omitting key factors that may bias results) [36, 37]. For studies of health-service use, reviewers should also consider whether analyses accounted for differences in clinical need between social groups to distinguish unfair inequalities/inequities from legitimate variation [38]. In cases where such data are not available, reviewers should adopt a qualitative or interpretive synthesis approach. This may involve thematic analysis-based comparisons across identity intersections, drawing on both narrative findings and contextual data. Particular attention should be paid to how primary studies explain observed differences, specifically whether they rely on behavioural, cultural or biological explanations, or whether they acknowledge structural determinants such as discrimination, racism, class inequality or exclusion from services [39]. Reviewers should also reflect on the depth of intersectionality in each study, considering whether it is used merely as a demographic descriptor or whether it meaningfully informs the study’s theoretical framework and analytical approach.

#### Life-course analysis

Reviewers should consider incorporating a life-course perspective to examine how exposures, interventions and outcomes are distributed, sequenced and accumulated across different life stages. Syntheses can be structured to identify whether and how evidence addresses critical periods (e.g. early childhood), cumulative disadvantage (e.g. long-term poverty), or transitional stages (e.g. entry to the labour market, retirement). This can be operationalised by coding for the life stage targeted by each study and mapping outcomes accordingly [33]. Where data permit, reviewers may apply age-stratified meta-analyses, trajectory modelling or synthetic cohort approaches to examine when inequalities emerge, persist or widen over time [33]. Life-course models such as the critical period framework, which emphasises the lasting impact of early-life exposures, and the accumulation model, which highlights the additive effects of repeated or prolonged disadvantage across time, can guide interpretation by highlighting the long-term impact of early exposures and the additive effects of disadvantage, respectively [15]. Reviewers should also reflect on evidence gaps in terms of temporal coverage. If the evidence base is disproportionately concentrated in early childhood and late life, with minimal attention to mid-life or transitional periods, this may limit the applicability of findings to cumulative or long-term policy responses.

## Synthesis stage

Reviewers should bring together quantitative and qualitative evidence using an inequality/inequity equity lens, moving beyond description to explore the mechanisms and contexts behind observed differences [40, 41]. Where data allow, they should assess how consistent and plausible these patterns are across social intersections and settings, noting when limited data or reporting restrict interpretation [42]. Confidence in findings should be judged transparently using appropriate tools, such as Grading of Recommendations Assessment, Development and Evaluation (GRADE) or GRADE-Confidence in the Evidence from Reviews of Qualitative Research (GRADE-CERQual) with inequality and/or inequity considerations [43, 44]. Finally, reviewers should draw clear, inequality/inequity-focused conclusions, distinguish structural causes from methodological artefacts, and highlight key evidence gaps for future research [41].

### Application of the Kunonga framework: a case example

To demonstrate the application of the Kunonga Framework, we present a case study on its integration into the conduct of a rapid systematic review on ethnic inequalities in palliative care prescribing in high-income countries [45]. The aim of the review was to explore ethnic inequalities in palliative care prescribing amongst adults residing in high-income countries [45]. Although the review protocol had been registered while the Kunonga Framework was still under development, its conceptual components were used to inform the review’s design, data extraction, analysis and interpretation. This allowed us to assess the feasibility and added value of applying the framework in practice. Table 3 summarises how the Kunonga Framework was operationalised, highlighting key insights and methodological challenges encountered during the review process.

**Table 3:** Application of the Kunonga framework in a review of ethnic inequalities in palliative prescribing [45]

Framework Stage	Component	Application in Case Study	Insights and Challenges
Protocol	Define Key Terms and Scope	The review focused on health inequalities, defined as observable differences in palliative prescribing among ethnic groups	The framework clarified the conceptual distinction between inequality and inequity. Retrospective reflection revealed how explicit definition at protocol stage improves alignment between aims, eligibility criteria, and analytical depth
	Frameworks and Theoretical Approach	PROGRESS-Plus was adopted post protocol registration to guide identification of relevant social stratifiers in data extraction and analysis [12]	The Kunonga Framework encouraged inclusion of life-course and intersectional perspectives, but original protocol did not anticipate these
	Logic Model	A logic model was developed post protocol to clarify hypothesised pathways linking social determinants (e.g. ethnicity, age) to prescribing outcomes. Moderators from PROGRESS-Plus were included [12]	Despite being developed post hoc, the model helped make explicit the mechanisms and assumptions underlying observed inequalities
Data Extraction	Mapping Data	Data extraction was reorganised to reflect intersecting demographic and social variables (e.g. age, gender, SES, place of residence, ethnicity), and life-course elements such as age at diagnosis	The framework supported a more unique categorisation of data, but limitations in primary study reporting constrained the extent of meaningful intersectional or temporal analysis
Analysis	Intersectionality	Analysis considered multiple PROGRESS-Plus factors (e.g. ethnicity, income, age) [12], using both adjusted regression models and subgroup comparisons where available	The framework revealed analytical gaps: few studies tested for interaction effects or explored intersecting inequalities. Narrative synthesis and author commentary were used to supplement quantitative findings
	Life-course Perspective	Limited application—only one study explored age-specific patterns in prescribing. Most lacked data to assess how prescribing varied over the life course or in relation to illness progression	The framework exposed significant gaps in temporal reporting. Its application highlighted the need for longitudinal or stage-specific data to enable assessment of cumulative disadvantage or life-stage-specific barriers
Synthesis	Integrating Findings	The synthesis examined how structural and contextual factors shaped ethnic inequalities in palliative prescribing, drawing on study authors’ explanations and wider evidence	The framework supported an inequality-focused synthesis combining quantitative and qualitative insights to explain intersecting disadvantages. Limited disaggregation and longitudinal data restricted understanding of cumulative disadvantage

Key PROGRESS-Plus: Place of residence, Race/ethnicity, Occupation, Gender, Religion, Education, Socioeconomic status, Social capital, plus additional factors such as personal characteristics, features of relationships, and time-dependent relationships

### Protocol stage

#### Define key terms and scope

The review focused on health inequalities rather than inequities because we were specifically interested in identifying and describing measurable differences in palliative care prescribing among ethnic groups. Health inequality was defined as observable differences, such as variations in medication type, dosage, or frequency of pain relief, without assuming these differences were unfair or avoidable [45]. This focus aligned with the review’s aim to document variations in prescribing practices in a way that was empirically grounded and descriptively clear [45]. Assessing inequities would require a different evidentiary and interpretive approach, incorporating ethical and contextual analysis that was beyond the scope of this review.

#### Frameworks and theoretical approach selection

The review adopted the PROGRESS-Plus framework after protocol registration to identify and extract factors relevant to prescribing inequality [12]. It offered a pragmatic and transferable structure for identifying key factors relevant to inequality and supported a systematic approach to data extraction.

#### Developing a logic model

**Fig. 1:**
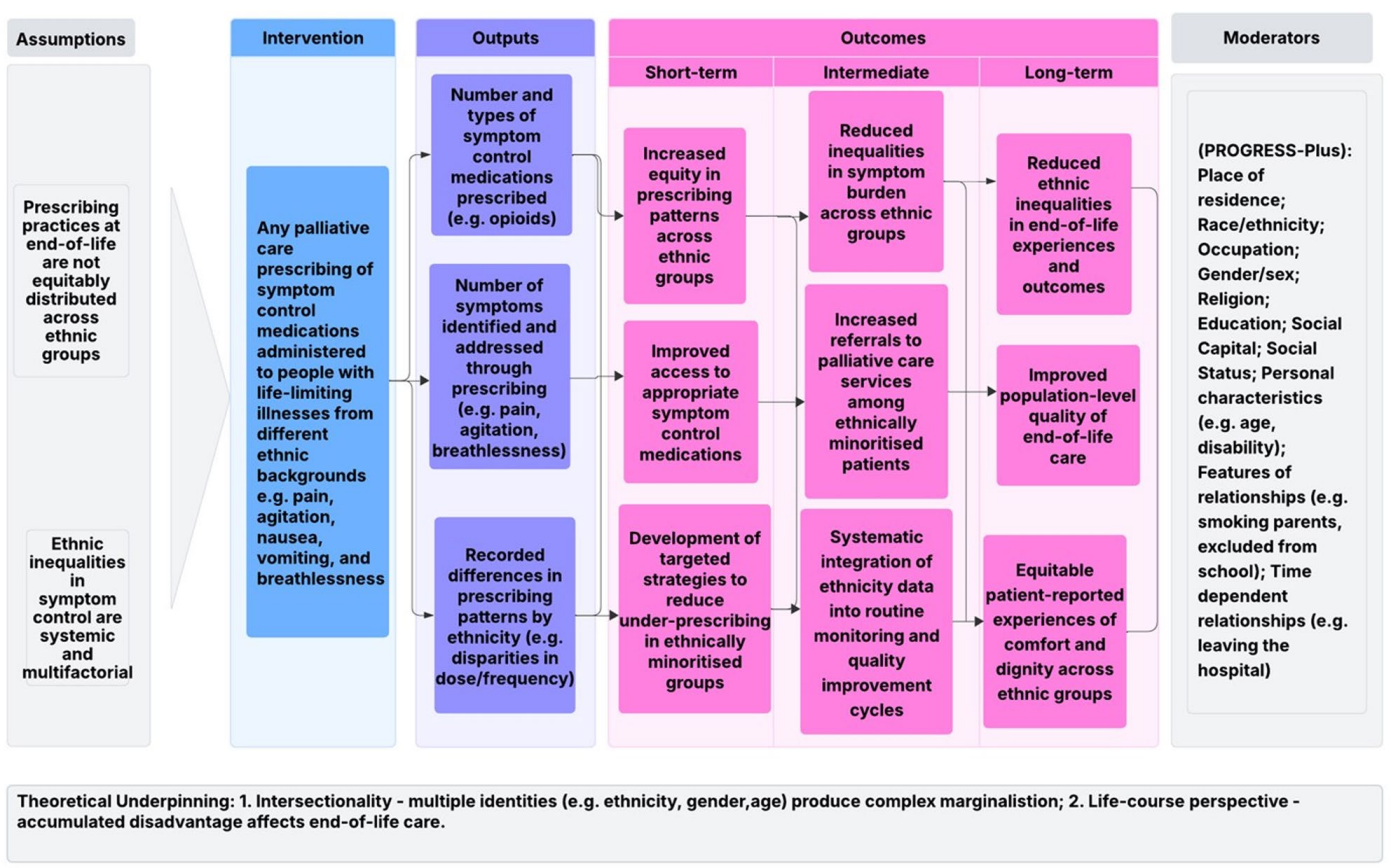
Logic model of ethnic inequalities in palliative prescribing: integrating intersectionality and life-course perspectives

A logic model was developed to clarify hypothesised pathways linking palliative prescribing practices to inequality-relevant outcomes (Fig. 1). The model articulated key assumptions, intervention components and expected short-, medium- and long-term outcomes. Moderating factors were drawn from PROGRESS-Plus factors to highlight how social position and identity-based disadvantage might influence prescribing patterns and symptom management [12].

### Data extraction stage

Data were extracted using a structured Excel form capturing study characteristics, participant demographics, prescribing practices and reported moderating factors. Guided by the Kunonga Framework, the extraction process was designed to highlight intersecting social and demographic factors relevant to health inequalities, including age, gender, socioeconomic status, place of residence and ethnicity. The data were also organised to capture life-course dimensions, such as age at diagnosis and experiences of persistent poverty [45].

### Analysis stage

#### Intersectionality

Applying an intersectional lens during analysis was challenging due to limited data disaggregation within the included studies. However, we systematically examined how multiple PROGRESS-Plus factors were addressed individually and in relation to ethnicity [12, 45]. In addition to extracting disaggregated findings, we assessed whether studies used statistical approaches that accounted for multiple social determinants simultaneously. This included identifying the use of multivariable regression models that adjusted for intersecting factors (e.g. ethnicity, income, age) in the analysis of access to palliative care or symptom management [45]. While these models did not always test for interaction effects explicitly, they offered partial insights into how multiple axes of inequality were considered concurrently. Where studies used stratified analysis or subgroup comparisons (e.g. by ethnicity or deprivation status), we examined whether findings varied systematically across these groups [45]. However, only a minority of studies explicitly considered the interplay between demographic factors or reported subgroup-specific outcomes in a way that allowed for intersectional interpretation.

#### Life-course analysis

Incorporating a life-course perspective into the analysis was similarly constrained by the limitations of the primary studies. Only one study reported age-specific trajectories or explored how prescribing practices varied across the stages of ageing, chronic illness progression or proximity to end of life. This lack of temporal granularity limited our ability to assess whether observed inequalities reflected cumulative disadvantage or stage-specific barriers to care [45].

#### Synthesis stage

The synthesis went beyond describing statistical results to consider how structural and contextual factors shaped the observed ethnic inequalities in palliative care prescribing [45]. Using study authors’ explanations and wider evidence, we explored possible mechanisms such as access barriers, discrimination, and socioeconomic disadvantage. Where quantitative findings were limited, qualitative insights helped explain how intersecting disadvantages influenced care [45]. We also noted ongoing evidence gaps, particularly poor disaggregation and limited longitudinal data, which restrict understanding of cumulative disadvantage and highlight priorities for improving primary research design and reporting [45].

## Discussion

### Main findings

This study introduces the Kunonga Framework as a structured, operational approach to embedding inequality and/or inequity-sensitive principles, specifically intersectionality and life-course theory, into evidence synthesis. While the theories are well-established in public health and social science literature [15, 30, 46], their practical application within evidence synthesis has remained inconsistent and underdeveloped. The Framework addresses key methodological gaps that were first identified in a prior systematic review [9], and subsequently explored through expert discussions. These gaps include the persistent conflation of health inequality and inequity, the limited integration of intersectionality due to insufficient data disaggregation and reliance on single-axis analyses, and the underuse of life-course perspectives, often constrained by a lack of temporally sensitive or longitudinal data [3, 33, 47, 48]. The framework offers practical guidance across the synthesis process, from clarifying conceptual definitions and selecting inequality and/or inequity-focused tools at the protocol stage (e.g. the SDOH, PROGRESS-Plus, HEIA, or the EQUALSS GUIDE Multiple framework), to supporting the identification of demographic and social factors and extracting and analysing data through an intersectional and life-course lens [10–12, 14].

### Added value of the Kunonga framework

Existing methodological resources such as the Cochrane Handbook and JBI Manual for Evidence Synthesis provide comprehensive guidance on systematic review methods across all stages [1, 49]. However, neither resource systematically integrates intersectionality or life-course perspectives into these processes. This gap in operationalising inequality and/or inequity theories beyond standard stratified “group” analysis underpins the focus of the Kunonga Framework on stages identified through expert consultation as having the greatest methodological gaps in inequality and/or inequity integration: protocol development, data extraction, analysis, and synthesis. The Kunonga Framework complements this existing guidance by filling that gap. It translates inequality and/or inequity theory into operational tools and analytic prompts that support reviewers to examine intersecting social factors and life-course influences in later stages of the review, where these dimensions most directly affect interpretation and synthesis.

### Strengths and limitations

This study used a structured, three-phase approach to develop the Kunonga Framework, integrating inequality and inequity considerations into evidence synthesis. The framework’s construction followed an iterative process: a systematic review to identify gaps; key informant discussions with eight health inequality and inequity experts; and synthesis of findings into a practical framework. This phased design allowed for continual refinement, ensuring that each phase built upon previous insights to produce a comprehensive, context-sensitive framework.

One key strength of this approach is its methodological rigour. The systematic review phase established a solid foundation by identifying gaps in existing inequality/inequity-focused evidence synthesis methodologies, ensuring that the framework built upon, rather than duplicated, prior work [9]. The use of key informant discussions provided qualitative insights, contextualising these gaps and guiding framework elements by capturing unique perspectives on the needs and challenges of inequality and/or inequity-focused syntheses [17, 18]. This combined approach facilitated a holistic understanding of the multi-dimensional factors influencing health inequalities, which is crucial for developing effective interventions. The use of an adapted framework analysis allowed us to work from a priori concepts while remaining open to emergent themes. By structuring the analysis around stages of the evidence synthesis process (protocol development, data extraction, analysis and synthesis), the framework offers clear guidance for researchers seeking to embed inequality and/or inequity equity considerations in a consistent manner.

However, there are limitations in the process of constructing the framework. First, the framework’s reliance on a limited number of experts means it may not fully represent the diverse perspectives and contexts relevant to global health inequities [50, 51]. While this offered depth in terms of specific expertise, the exclusion of patients, public contributors and voluntary or charitable organisations constrained the diversity of voices and experiential knowledge captured [25]. Including lived experience perspectives may have revealed different priorities, inequality and/or inequity concerns or framings, particularly around what counts as meaningful inequalities or inequities, or how intersecting disadvantage can manifest. Second, while the framework was tested using a single case study, it has not yet been implemented across diverse review designs or topics. Third, the current iteration of the Kunonga Framework does not include detailed guidance for searching and screening. Expert consultation indicated that the most underdeveloped and consequential gaps in intersectionality and life-course integration lie in protocol development, data extraction, analysis, and synthesis. However, we acknowledge that while equity considerations are discussed in existing searching and screening guidance (e.g. Cochrane Handbook, JBI Manual), these resources do not embed intersectionality or life-course approaches within those processes [1, 49]. Developing approaches that explicitly incorporate intersectionality and life-course considerations into search strategies, eligibility criteria, and screening judgements is therefore a priority for future iterations of the framework. In addition, the primary focus was on the methodological and analytical dimensions of inequality and/or inequity, rather than on the evaluation of interventions themselves. However, we recognise that understanding what interventions aim to achieve, how they are expected to bring about change through a programme theory or theory of change, and the contexts in which they operate, are all central to interpreting inequality and/or inequity impacts and translating findings into policy [52–54]. These areas were beyond the scope of our current work and were not explicitly explored during the framework’s development but represent important directions for future refinement and empirical testing. Lastly, the framework’s practical application is constrained by the quality of the underlying evidence base. Despite repeated calls for improvement, most primary studies still lack disaggregated reporting by key social stratifiers and longitudinal follow-up, perpetuating long-recognised evidence gaps in health inequality and/or inequity research [55]. These limitations lie upstream of evidence synthesis and are not limitations of the Kunonga Framework itself or its development process. However, they do restrict the extent to which inequality and/or inequity can be explored in practice. The framework offers a structured means of documenting and exposing such data gaps, thereby supporting calls for improved primary research design and reporting standards.

### Implications for research

The development of the Kunonga Framework marks a step toward operationalising inequality and/or inequity-sensitive methodologies in evidence synthesis. However, its introduction also highlights several critical areas for future research. First, while the framework offers structured guidance for integrating intersectionality and life-course perspectives, its practical application remains underexplored across diverse evidence synthesis types and health contexts. Future studies should prospectively apply the framework to assess its adaptability, usability and impact on review outcomes. Second, the framework’s emphasis on intersectionality and life-course theory exposes persistent limitations in the primary literature, particularly the lack of disaggregated and longitudinal data. These gaps constrain the ability of reviewers to conduct unique analyses and highlight the need for upstream improvements in study design and reporting. Research funders and institutions should prioritise data infrastructure that supports inequality and/or inequity-focused synthesis, including the routine collection of sociodemographic variables and life-course indicators. Wider adoption of reporting extensions such as Preferred Reporting Items for Systematic Reviews and Meta-Analyses (PRISMA)-Equity and Strengthening the Reporting of Observational Studies in Epidemiology (STROBE)-Equity can further enhance the feasibility of applying frameworks like Kunonga in practice [56, 57]. Third, the framework was developed mainly for quantitative and mixed-method evidence syntheses, where challenges in addressing inequality and inequity are most evident. Its core principles may also inform qualitative and realist reviews but applying them in these contexts will require further adaptation and testing in future work. We also recognise the ongoing debate about whether intersectionality can or should be examined using quantitative methods. Some scholars argue that statistical interactions risk oversimplifying its structural and theoretical roots [58, 59]. Others contend that, when guided by theory and complemented by qualitative insights, quantitative approaches such as MAIHDA can still provide valuable evidence on intersecting disadvantages [42, 60]. Fourth, although the Kunonga Framework was developed mainly for reviews that focus on health inequality and/or inequity, its principles can also be useful in other types of evidence syntheses that examine differences between groups, settings, or changes over time. In such cases, reviewers may use selected components of the framework to enhance transparency and interpretation without altering the review’s main objectives. Finally, the application of the Kunonga Framework to a rapid review on ethnic inequalities in palliative care prescribing provides an initial demonstration of its feasibility and added value [45]. The case study illustrates how the framework can enhance conceptual clarity, guide data extraction and support intersectional and temporal analyses, even when applied following protocol registration. Future research should build on this example by embedding the framework prospectively in review protocols, thereby strengthening its empirical foundation and refining its components through iterative testing.

### Implications for policy

The Kunonga Framework will not only enhance our comprehension of health inequalities or inequities within evidence syntheses but will also offer a pathway for translating research findings into actionable policy recommendations and interventions. By identifying the root causes of health inequalities/inequities and understanding their dynamic nature across the life-course and among diverse populations, policymakers and practitioners will be better able to design targeted interventions that address underlying structural factors, but also to make decisions that are informed by a clearer understanding of potential inequalities and/or inequities.

## Conclusion

This paper contends that the current state of evidence synthesis in health inequality and inequity research is inadequate to capture the multifaceted and dynamic nature of the issue. The Kunonga Framework offers a structured, theory-informed approach for integrating health inequality and/or inequity considerations across protocol development, data extraction, analysis, and synthesis. By embedding principles of intersectionality and the life-course perspective, it aims to move beyond surface-level subgroup analysis and support inequality and/or inequity-sensitive methodology in practice.

Although the framework was developed to support inequality and/or inequity-focused reviews, its components are designed to be useful in broader evidence syntheses where variation across populations, contexts, or stages of life may influence how interventions work. As inequality and inequity remain pressing concerns for health and care policy, the Kunonga Framework can help reviewers make analyses more inclusive, reflective, and decision relevant. Future research should test the framework prospectively across different review designs and topics, assess its use with both qualitative and quantitative methods, and develop searching and screening methods that explicitly embed intersectionality and life-course principles.

## Supplementary Information

Supplementary file 1. Consolidated Criteria for Reporting Qualitative Research (COREQ) checklist.

Supplementary file 2. Key Informant Discussions Agenda.

Supplementary file 3. Analysis Coding Structure.

Supplementary file 4. Intersectionality- informed data extraction template for evidence synthesis.

## Data Availability

The datasets generated and/or analysed during the current study are not publicly available due to confidentiality agreements with expert participants but are available from the corresponding author on reasonable request.

## Abbreviations

COREQ: Consolidated Criteria for Reporting Qualitative Research
EQUALSS GUIDE Multiple framework: Ethnicity and race, Qualifications and education, Underserved area, Age, Language and religion, Sex, Sexual orientation, Gender identification, Underrepresented groups (inclusion groups), Income and wealth, Disability (physical, mental and learning), Employment and occupation, and Multiple disadvantages
GRADE: Grading of Recommendations Assessment, Development and Evaluation
GRADE-CERQual: Grading of Recommendations Assessment, Development and Evaluation-Confidence in the Evidence from Reviews of Qualitative Research
HEIA: Health Equity Impact Assessment
IPD: Individual Patient Data
MAIHDA: Multilevel Analysis of Individual Heterogeneity and Discriminatory Accuracy
NIHR-INCLUDE: National Institute for Health and Care Research Innovations in Clinical Trial Design and Delivery for the Under-served
PRISMA Equity: Preferred Reporting Items for Systematic Reviews and Meta-Analyses-Equity
PROGRESS Plus: Place of residence, Race/ethnicity, Occupation, Gender, Religion, Education, Socioeconomic status, Social capital, plus additional factors such as personal characteristics, features of relationships, and time-dependent relationships
SDOH: Social Determinants of Health
STROBE: Strengthening the Reporting of Observational Studies in Epidemiology

## Footnotes

1 PROGRESS-Plus: Place of residence, Race/ethnicity/culture/language, Occupation, Gender/sex, Religion, Education, Socioeconomic status, and Social capital, plus additional factors such as personal characteristics (e.g., age, disability), relational influences (e.g. smoking parents), and time-dependent variables (e.g. hospitalisation).

2 EQUALSS GUIDE: Ethnicity and race, Qualifications and education, Underserved area, Age, Language and religion, Sex, Sexual orientation, Gender identification, Underrepresented groups (inclusion groups), Income and wealth, Disability (physical, mental and learning), Employment and occupation, and Multiple disadvantages.
